# Marburg Virus Structure Revealed in Detail

**DOI:** 10.1371/journal.pbio.1001198

**Published:** 2011-11-15

**Authors:** Richard Robinson

**Affiliations:** Freelance Science Writer, Sherborn, Massachusetts, United States of America

## Abstract

Ultrastructural analysis of a filovirus assembling within infected eukaryotic cells reveals differences in structure and assembly mechanisms between related RNA viruses.

**Figure:**
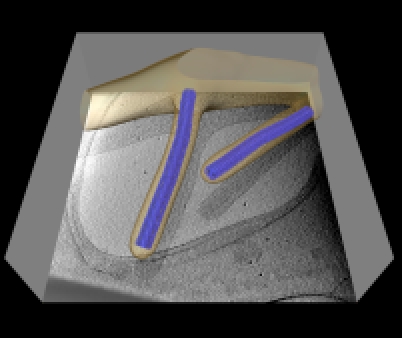
Marburg virus particles were imaged in 3-D in the act of budding from infected cells. Analyzing such images sheds light on the structure of the virus and the mechanisms by which it is assembled.

When it comes to nasty pathogens, Marburg virus is among the nastiest. Cousin to Ebola virus, Marburg causes fever, rash, delirium, and severe hemorrhaging, often ending in organ failure and death. It is rare in the wild, but was a central focus of weaponization by the Soviet Union, and remains a concern for terrorism experts who fear its lethal potential and resistance to treatment.

One reason that treatments have proved so elusive is because the virus is so hard to work with—hazmat suits, self-contained breathing gear, and electronically secured airlocks are all required for even the simplest of studies with live virus. But another reason is that the virion (the viral particle) is heterogeneous in shape, and that heterogeneity has confounded standard imaging techniques (X-ray crystallography, cryo-electron microscopy [cryoEM]), which require purified, identical particles to obtain their highest resolution.

In this issue of *PLoS Biology*, Tanmay Bharat, John Briggs, and colleagues get around that problem by using a sophisticated combination of imaging techniques that, through multiple iterations, provides the first clear three-dimensional picture of the intact Marburg virion structure.

Marburg and Ebola are the only two identified viruses that make up the small family of filoviruses, a relative of the larger family of rhabdoviruses. Both families contain an RNA strand coated with proteins, but the rhabdoviruses are more structurally homogenous, which makes them easier to study. The prototypical rhabdovirus is vesicular stomatitis virus (VSV), and is the only member of the family for which detailed information is available on structure and host–cell interactions. But it has not been clear how broadly insights from VSV can be applied to related viruses.

The authors began by isolating Marburg virions from infected cells, and imaging them using cryoEM, a technique that freezes samples to prevent their distortion in the evacuated atmosphere of the electron microscope. The virion occurred in three forms: filamentous, round, and a combination particle shaped like the number 6 (these three forms likely represent a continuum of possible virion shapes). In the next step, using the same principle as a CAT scanner, they collected many images taken at different angles, and processed them to create three-dimensional images of the virions.

Turning their attention to the filamentous particle, they found it was composed of concentric layers of different density, which they suspected corresponded to the different proteins known to make up the virion. To determine which was located where, they sectioned the virions and stained them with antibodies to each of the known proteins in succession, then imaged them in the electron microscope. (Gold particles attached to the antibodies show up as jet black dots in the EM). Their work allowed them to assign one or more proteins to each layer, with the viral RNA linked to a protein at the center. They further confirmed their results by purifying the RNA-binding protein (nucleoprotein) alone, which could be imaged in the cryoEM.

The core of the filament, called the nucleocapsid, was studded with boomerang-shaped protrusions that spiraled around the outside, composed of two different viral proteins, and an inner, more dense set of lobes, each of which contained the RNA-nucleoprotein complex. The average nucleocapsid contained over 3,000 copies of the nucleoprotein, arranged in a helix and each binding to six RNA bases of the viral genome (in contrast, VSV nucleoprotein binds nine RNA bases).

The viral life cycle requires nucleocapsids to bud from the host cell membrane to form the infectious particle. Using cryoEM, the authors found that the host cell's filopdia, membrane extensions from which the virus buds, could contain more than one nucleocapsid. The nucleocapsid has a directionality, with the “pointed” end always being first to be enveloped by host cell membrane, and the “barbed” end following. In contrast, VSV buds with its barbed end first. In that virus, the earliest synthesized part of the genome, the so-called three-prime end, is packed in the barbed end, while in a related virus, respiratory syncytial virus (RSV), it is in the pointed end. If Marburg virus orients its RNA as RSV does, the three-prime end of its genome would also be the first to bud, as it is in VSV.

The results presented are the first detailed three-dimensional look at the structure of the Marburg virus, and thus provide a wealth of new information that will likely lead to a better understanding of the virus's lethal biology. It should also illuminate aspects of related viruses, including Ebola and rabies, which also bear single-stranded RNA genomes and have many similar structural features, but differ in some fundamental details of their life cycles.

**Bharat TAM, Riches JD, Kolesnikova L, Welsch S, Krähling V, et al. (2011) Cryo-Electron Tomography of Marburg Virus Particles and Their Morphogenesis within Infected Cells. doi:10.1371/journal.pbio.1001196**

